# Smoking Intensity Increases Diaphragm Muscle Injury: A Clinicopathologic Study

**DOI:** 10.3390/jcm12113823

**Published:** 2023-06-02

**Authors:** Ricardo Aparecido Baptista Nucci, Alexandre Leopold Busse, Romeu Rodrigues de Souza, Laura Beatriz Mesiano Maifrino, Carlos Augusto Pasqualucci, Carlos Alberto Anaruma, Renata Elaine Paraizo Leite, Roberta Diehl Rodriguez, Claudia Kimie Suemoto, Wilson Jacob-Filho

**Affiliations:** 1 Department of Pathology, Faculty of Medicine, University of São Paulo, São Paulo 01246-903, Brazil; alebusse@gmail.com (A.L.B.); cpasqua@usp.br (C.A.P.); robertadiehlr@gmail.com (R.D.R.); wiljac@usp.br (W.J.-F.); 2 Laboratory of Medical Research in Aging (LIM-66), Division of Geriatrics, Faculty of Medicine, University of São Paulo, São Paulo 01246-903, Brazil; cksuemoto@usp.br; 3 Department of Anatomy, Institute of Biomedical Sciences, University of São Paulo, São Paulo 05508-000, Brazil; souzarrd@uol.com.br; 4 Department of Anatomy, 9 de Julho University—UNINOVE, São Paulo 03155-000, Brazil; 5 Laboratory of Clinical Analysis, Faculty of Medicine of the ABC District, Santo André 09060-650, Brazil; lmaifrino@uol.com.br; 6 Dante Pazzanese Institute of Cardiology, São Paulo 04012-909, Brazil; 7 Laboratory of Morphology and Physical Activity, Department of Physical Education, São Paulo State University “Júlio de Mesquita Filho”, Rio Claro 11330-900, Brazil; carlos.anaruma@unesp.br; 8 Laboratory of Physiopathology in Aging (LIM-22), Department of Pathology, Faculty of Medicine, University of Sao Paulo, São Paulo 01246-903, Brazil; renataparaizoleite@gmail.com

**Keywords:** autopsy, cigarette, diaphragm, histopathology

## Abstract

Background: Studying the effects of smoking intensity is important to evaluate the risk of tobacco use on a range of illnesses, such as as sarcopenia among the elderly. Thus, this study aimed to analyze the effects of pack-years of cigarette smoking on the diaphragm muscle (DIAm) histopathology of postmortem samples. Methods: Subjects were divided into three groups: never-smoker (*n* = 46); less than 30 pack-years of smoking (*n* = 12); and more than 30 pack-years of smoking (*n* = 30). Diaphragm samples were stained with Picrosirius red and hematoxylin and eosin stain for general structure. Results: Participants with more than 30 pack-years of cigarette smoking had a significant increase in adipocytes, blood vessels and collagen deposit, as well as an increase in histopathological alterations. Conclusions: Pack-years of smoking was associated with DIAm injury. However, further clinicopathological studies are needed to confirm our findings.

## 1. Introduction

During the 20th century, cigarette smoking became the predominant socially acceptable addiction, which has led to a global lung cancer epidemic [1]. While the prevalence of smoking has decreased in several high-income countries, many low-income and middle-income countries continue to experience an upward trend [2]. Thus, to understand the effects of smoking on different biological systems is of paramount importance to develop health-promoting strategies to reduce the burden of smoking-related diseases. In this scenario, studies highlighted that smoking is a major risk factor for several diseases, including sarcopenia [3,4,5,6,7].

A history of cigarette smoking, which ranges from patients who smoke a few cigarettes a day for a few years to patients who smoke packs of cigarettes daily for decades, is a parameter of major importance to understand the severity of smoking-related diseases [8,9,10,11,12]. Therefore, the measures of intensity (pack-years) and duration of smoking are essential to establish pathological relations.

Although studies focus to understand the effects of smoking on lung morphology, recent studies have shown that even the diaphragm musculature is injured by tobacco use [13,14,15,16]. The diaphragm muscle (DIAm) is a major responsible musculature for inspiratory behavior, which is negatively affected by advancing age [17,18], respiratory diseases (e.g., COPD) [19], and smoking [6,13,14,15,16]. Cigarette smoking seems to increase DIAm inflammation, leading to fiber injury and fibrosis [15,16]. Thus, comprehending the effects of smoking intensity on the DIAm may shed some light onto new therapeutic approaches.

Therefore, we aimed to analyze the effects of cigarette-smoking intensity (pack-years) on the DIAm of postmortem samples.

## 2. Materials and Methods

### 2.1. Study Protocol

This study was approved by the Faculty of Medicine of the University of São Paulo (ethical approval number 2.209.383/2017) and was in accordance with the Helsinki Declaration. Data were collected from January 2019 to September 2019. As previously described, a semi-structured interview was conducted with the subject’s next of kin [15,16,17]. We included individuals with no respiratory pathologies (smokers and non-smokers) and we excluded subjects with conditions that negatively affect the musculature, as previously described [15,16,17].

Variables of interest included age, sex, body mass index (BMI), smoking status, cardiovascular cause of death, diabetes mellitus, hypertension, stroke, muscle abnormalities, blood vessels, fat and collagen deposition.

### 2.2. Smoking Intensity (Pack-Years)

The smoking subjects were past-smokers (quit smoking 1 year before death) and current-smokers (smoked 1 week before death) as previously categorized [12,20,21]. A cut-off value of 30 pack-years (1 pack-year = 20 cigarettes per day for 1 year) was used to divide the smoking group in less than 30 pack-years of smoking and more than 30 pack-years of smoking for comparison [9,10,11,12,22].

### 2.3. Sample Collection and Histological Techniques

Samples were collected, fixed in formalin and embedded in paraffin, as previously described [15,16,17,23,24]. Transverse sections were stained with Picrosirius red for fibrosis and standard hematoxylin and eosin (H&E) for histopathological parameters [15,16,23,24]. Histopathology was analyzed by morphologists blinded to clinical data [25].

### 2.4. Histopathological Analysis

Abnormal fibers were analyzed as previously described [15,16,17,18,19] and classified into three categories: (a) central nucleus, defined as a minimum of 2 μm of cytoplasm between the nucleus and sarcolemma, indicating muscle wasting; (b) abnormal size and shape, which included small fibers with less than one-third the diameter of the three largest fibers in the field, indicating atrophy; and (c) abnormal cytoplasm, characterized by vacuolated or ruptured fibers, indicating loss of cytoplasmic integrity and typically surrounded by inflammatory infiltrates. Additionally, we analyzed the volume density of adipocytes, blood vessels and collagen fibers. We used stereology to analyze the volume density of each parameter with the aid of ImageJ software (version 1.47, National Institutes of Health) as previously described [15,16,17]. Using morphometric methods, we analyzed the area (μm^2^) of 20 normal muscle fibers per subject using Axio Vision software (Zeiss, Jena, Germany).

### 2.5. Statistical Analysis

The mean ± standard error (SEM) and absolute or relative frequencies were used to express values for continuous and categorical variables, respectively. Continuous variables were compared between groups using a one-way ANOVA with Newman–Keulst post-test, while a two-tailed unpaired Student’s t-test was used to compare smokers based on ‘years of smoking’ and ‘pack-years’. Chi-square or Fisher exact tests were used as appropriate. To assess the association between smoking intensity (pack-years) and histopathological findings, a multivariate linear regression was performed and adjusted for age, sex, body mass index, cardiovascular cause of death, diabetes mellitus, hypertension and stroke. The beta coefficient (β) and 95% confidence interval (95% CI) were used to report the results. The statistical analysis was conducted using SPSS software (IBM Statistics version 21.0) with an alpha level set at 0.05.

## 3. Results

A total of 120 subjects were selected. We excluded thirty-two participants, of which twenty-seven had respiratory diseases, such as asthma and chronic obstructive pulmonary disease, and five participants had metastasis on DIAm. Thus, 88 individuals were eligible for the study; 53% were male. In Table 1, we can see that compared to non-smoker participants, the subjects with more than 30 pack-years of smoking were significantly younger (*p* < 0.0007).

**Figure 1:**
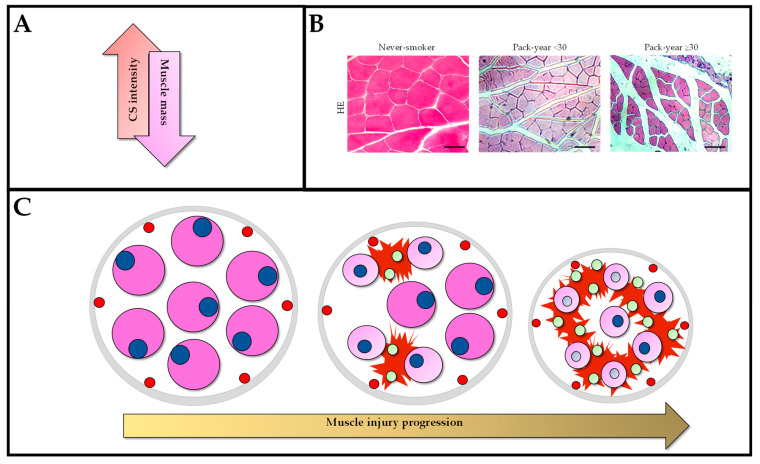
Schematic representation of the injury caused by smoking intensity (pack-years). (**A**) With increasing smoking load, the production of muscle maintenance-related proteins decreases, leading to a reduction in muscle mass. (**B**) Illustrative photomicrographs of the reduction in a cross-sectional area of muscle fibers (scale bar = 50 µm). (**C**) Progression of DIAm injury due to smoking intensity, where there is a decrease in the cross-sectional area of the diaphragm muscle, followed by migration of the peripheral nucleus to a central nucleus, increased inflammatory infiltrates (seen in green) that may lead to degradation of muscle fibers (ruptured cytoplasm) and increased fibrosis (seen in red). We can see capillaries in the periphery (red with black contour).

The H&E technique showed that muscle injury were present in both smokers and control subjects, but were increased in smoker subjects (Figure 1). We observed that an increase in histopathological features was associated with pack-years of smoking (Table 1) and a linear regression analysis highlighted the association between pack-years of smoking and the histopathological characteristics analyzed (Table 2). In respect to abnormal size and shape, we observed a significant increase of 71.90% (*p* < 0.0001) in subjects with ≥30 pack-years of smoking when compared to non-smokers. Further, we highlight that subjects with ≥30 pack-years of smoking had a significant increase of 335.95% (*p* < 0.0001) in abnormal cytoplasm when compared to non-smokers. In the adipocyte deposition analysis, we observed that subjects with ≥30 pack-years of smoking had a significant increase of 83.52% (*p* < 0.0001) when compared to non-smokers. In addition, we observed a significantly sharp increase of 450% (*p* < 0.0001) of blood vessels’ volume densities in subjects with ≥30 pack-years of smoking when compared to non-smokers. Additionally, we observed a significant increase of 63.88% (*p* < 0.0001) in the central nucleus of ≥30 pack-years of smoking when compared to participants that never smoked.

The group with ≥30 pack-years of smoking had an increase of 39.98% (*p* < 0.0001) of collagen fibers compared to the never-smoked group. However, the cross-sectional area of normal myofibers had a significant decrease of 11.76% (*p* < 0.0001) in participants with ≥30 pack-years of smoking when compared to non-smokers.

## 4. Discussion

We suggested that DIAm injury was related to smoking intensity in an autopsy study of Brazilian individuals. Studying the cumulative effects of smoking is important to evaluate the risk of tobacco use on a range of illnesses, such as sarcopenia in the elderly [6,13,14]. Studies showed that cigarette smoking is associated with muscle protein breakdown and injury, followed by fibrosis, even in the DIAm [6,13,14,15]. Additionally, in a previous pilot study by our group, subjects with ≥30 pack-years of smoking had an increase in DIAm remodeling, inflammation, oxidative stress and vascularization compared to non-smokers [16], which are in accordance with our findings. The participants in our study showed a decrease in cross-sectional area, suggesting muscle wasting, followed by the migration of the peripheral nucleus to a central nucleus, increased degradation of muscle fibers and increased fibrosis as observed in studies with COPD smokers [23] and non-COPD smokers [15]. Additionally, we observed an increase in blood vessels in subjects with ≥30 pack-years of smoking as an adaptative response to avoid a hypoxic state [16,26].

When individuals smoke cigarettes, various components, including aldehydes, reactive oxygen species (ROS) and reactive nitrogen species (RNS), are released [27]. These substances are both volatile and soluble, allowing them to enter the bloodstream and reach the skeletal muscles of smokers. Once in the skeletal muscle, cigarette-smoking (CS) components increase oxidative stress either directly or by activating nicotinamide adenine dinucleotide phosphate (NADPH) oxidase (NOX), resulting in the production of ROS and increased oxidative stress [27]. CS-induced oxidative stress may lead to p38 MAPK phosphorylation, which activates the NF-kB pathway through IKK and IkB phosphorylation, and the proteasomal degradation of IkB. This results in the nuclear translocation of NF-kB, which upregulates muscle-specific E3 ubiquitin ligases [27]. Upregulation of these ligases leads to increased skeletal muscle protein degradation, thus accelerating the progression of sarcopenia in smokers. In this sense, the structural differences seen in our study can be related to this process.

Our study should be considered in relation to the study limitations. Several conditions such as the aging process could potentially affect the musculature [17,18]. Despite the age of the participants, significant differences were observed between the groups. Additionally, we did not analyze the results regarding cigarette brands or their isolated harmful components. Although we only used histochemical techniques to assess muscle histopathology, our results were consistent with the literature on evaluating DIAm histology in both healthy and ill individuals [15,16,17,23]. Nevertheless, future studies should add molecular and biomechanical techniques to analyze the effects of different smoking intensities on the DIAm.

However, it is imperative to acknowledge the strengths of this study. First, the study presented clinical and histopathological data of smokers without any signs of respiratory pathology. Second, a multivariate analysis was conducted to avoid any biases from confounding variables. Third, the morphological findings were homogeneous across all sites, as indicated by our standard deviation. This finding may be attributed to the prolonged smoking period of the included patients, which resulted in long-term injury to the DIAm. Finally, the parameters examined were consistent with the literature [15,16,17,18,19,23].

Awareness of the harmful effects of smoking on the body, including its potential to cause muscular injury, is critical for public health. Therefore, there is a pressing need to increase public awareness of these effects and promote smoking cessation to mitigate the risk of muscular injury and other health issues associated with smoking. Furthermore, we recommend that future clinical investigations incorporate respiratory exercises as a component of physiotherapeutic interventions to effectively enhance pulmonary ventilation and improve diaphragmatic strength in smokers who have experienced diaphragmatic injury as a result of tobacco smoking.

## 5. Conclusions

In conclusion, our findings suggest that there is a dose-dependent effect of smoking on diaphragmatic injury, as higher intensities of smoking were associated with increased injury in a sample of Brazilian individuals. Therefore, further clinicopathological studies incorporating molecular techniques are required to confirm our results.

## Figures and Tables

**Table 1:** Characteristics and histopathological analysis between the groups.

	Never-Smoker (*n* = 46)	Pack-Year < 30 (*n* = 12)	Pack-Year ≥ 30 (*n* = 30)
**Characteristics**			
Age (years), mean (SEM) ^1^	79.35 (2.00)	72.17 (3.45)	67.50 (2.18) ^a^
Male, n (%) ^2^	24 (52.17)	7 (58.33)	16 (53.33)
Weight (kg), mean (SEM) ^1^	60.45 (2.82)	57.87 (5.82)	66.11 (3.62)
BMI (kg/m^2^), mean (SEM) ^1^	20.94 (0.81)	20.99 (1.85)	22.43 (0.90)
Period of smoking (years), mean (SEM) ^3^	-	24.92 (4.15)	48.07 (2.22) ^b^
Pack-years of smoking, mean (SEM) ^3^	-	20.21 (1.97)	69.72 (5.56) ^b^
Cardiovascular cause of death, n (%) ^2^	21 (45.65)	6 (50)	18 (60)
Diabetes mellitus, n (%) ^2^	18 (39.13)	6 (50)	10 (33.33)
Hypertension, n (%) ^2^	25 (54.34)	7 (58.33)	15 (50)
Stroke, n (%) ^2^	5 (10.86)	3 (25)	4 (13.33)
**Histopathological analysis**			
Abnormal size and shape, mean (SEM) ^1^	2.10 (0.06)	2.85 (0.09) ^a^	3.61 (0.03) ^ab^
Abnormal cytoplasm, mean (SEM) ^1^	2.67 (0.09)	5.91 (0.37) ^a^	11.64 (0.49) ^ab^
Adipocyte deposition, mean (SEM) ^1^	3.25 (0.24)	6.33 (0.28) ^a^	8.47 (0.65) ^ab^
Blood vessels, mean (SEM) ^1^	0.40 (0.10)	1.60 (0.30) ^a^	2.20 (0.40) ^a^
Central nucleus, mean (SEM) ^1^	7.20 (0.50)	8.50 (0.75)	11.80 (0.25) ^ab^
Collagen fibers, mean (SEM) ^1^	13.78 (0.07)	15.92 (0.11) ^a^	19.29 (0.22) ^ab^
Cross-sectional area, mean (SEM) ^1^	1301 (2.85)	1227 (1.56) ^a^	1148 (3.49) ^ab^

BMI: Body mass index. SEM: Standard error of the mean. ^1^ One-way ANOVA (Newman–Keulst post hoc). ^2^ Chi-square test. ^3^ Unpaired *t*-test. ^a^ *p* < 0.05 vs. Never-smoker; ^b^ *p* < 0.05 vs. Pack-year <30.

**Table 2:** Association between pack-years of smoking and histopathological findings (*n* = 42).

	Model I	Model II
	β Coefficient (95% CI)	β Coefficient (95% CI)
Abnormal size and shape	0.0271 (0.0247; 0.0295) *	0.0553 (0.0511; 0.0594) *
Abnormal cytoplasm	0.0431 (0.0405; 0.0457) *	0.0853 (0.0789; 0.0918) *
Adipocyte deposition	0.0525 (0.0477; 0.0574) *	0.0523 (0.0472; 0.0575) *
Blood vessels	0.0139 (0.0132; 0.0147) *	0.0277 (0.0252; 0.0301) *
Central nucleus	0.0242 (0.0207; 0.0277) *	0.0529 (0.0483; 0.0575) *
Collagen fibers	0.0222 (0.0206; 0.0237) *	0.0459 (0.0430; 0.0488) *
Cross-sectional area	−0.6347 (−0.7190; −0.5505) *	−1.3764 (−1.4726; −1.2802) *

Model I: Linear regression analysis between pack-years of smoking and histopathological findings unadjusted for cofounding variables. Model II: Multivariate linear regression analysis between pack-years of smoking and histopathological findings adjusted for age, sex, body mass index, cardiovascular cause of death, diabetes mellitus, hypertension and stroke. * *p* < 0.01.

## Data Availability

All data generated or analyzed during this study are included in this article. Further enquiries can be directed to the corresponding author.

## References

[1] Doll R., Boyle P., Gray N., Henningfield J.E., Seffrin J., Zatonski W. (2010). Evolution of knowledge of the smoking epidemic. Tobacco Science, Policy, and Public Health.

[2] Pierce J.P., White V.M., Emery S.L. (2012). What public health strategies are needed to reduce smoking initiation?. Tob. Control.

[3] Nelson H.D., Nevitt M.C., Scott J.C., Stone K.L., Cummings S.R. (1994). Smoking, alcohol, and neuromuscular and physical function of older women. JAMA.

[4] Castillo E.M., Goodman-Gruen D., Kritz-Silverstein D., Morton D.J., Wingard D.L., Barrett-Connor E. (2003). Sarcopenia in elderly men and women: The Rancho Bernardo study. Am. J. Prev. Med..

[5] Szulc P., Duboeuf F., Marchand F., Delmas P.D. (2004). Hormonal and lifestyle determinants of appendicular skeletal muscle mass in men: The MINOS study. Am. J. Clin. Nutr..

[6] Petersen A.M.W., Magkos F., Atherton P., Selby A., Smith K., Rennie M.J., Pedersen B.K., Mittendorfer B. (2007). Smoking impairs muscle protein synthesis and increases the expression of myostatin and MAFbx in muscle. Am. J. Physiol. Endocrinol. Metab..

[7] Steffl M., Bohannon R.W., Petr M., Kohlikova E., Holmerova I. (2015). Relation between cigarette smoking and sarcopenia: Meta-analysis. Physiol. Res..

[8] Janjigian Y.Y., McDonnell K., Kris M.G., Shen R., Sima C.S., Bach P.B., Rizvi N.A., Riely G.J. (2010). Pack‐years of cigarette smoking as a prognostic factor in patients with stage IIIB/IV nonsmall cell lung cancer. Cancer.

[9] Fabricius P., Løkke A., Marott J.L., Vestbo J., Lange P. (2011). Prevalence of COPD in Copenhagen. Respir. Med..

[10] National Lung Screening Trial Research Team (2011). Reduced lung-cancer mortality with low-dose computed tomographic screening. N. Engl. J. Med..

[11] Saquib N., Stefanick M.L., Natarajan L., Pierce J.P. (2013). Mortality risk in former smokers with breast cancer: Pack‐years vs. smoking status. Int. J. Cancer.

[12] Wada M., Takeshima T., Nakamura Y., Nagasaka S., Kamesaki T., Kajii E., Kotani K. (2017). Association between smoking and the peripheral vestibular disorder: A retrospective cohort study. Sci. Rep..

[13] Barreiro E., Peinado V.I., Galdiz J.B., Ferrer E., Marin-Corral J., Sánchez F., Gea J., Barberà J.A. (2010). Cigarette smoke–induced oxidative stress: A role in chronic obstructive pulmonary disease skeletal muscle dysfunction. Am. J. Respir. Crit. Care Med..

[14] Barreiro E., del Puerto-Nevado L., Puig-Vilanova E., Pérez-Rial S., Sánchez F., Martínez-Galán L., Rivera S., Gea J., González-Mangado N., Peces-Barba G. (2012). Cigarette smoke-induced oxidative stress in skeletal muscles of mice. Respir. Physiol. Neurobiol..

[15] Nucci R.A.B., de Souza R.R., Suemoto C.K., Busse A.L., Maifrino L.B.M., Pasqualucci C.A., Anaruma C.A., Jacob-Filho W. (2019). Cigarette Smoking Impairs the Diaphragm Muscle Structure of Patients without Respiratory Pathologies: An Autopsy Study. Cell. Physiol. Biochem..

[16] Nucci R.A.B., Maifrino L.B.M., Busse A.L., De Souza R.R., Pasqualucci C.A., Anaruma C.A., Leite R.E.P., Rodriguez R.D., Suemoto C.K., Jacob-Filho W. (2020). Evaluation of the Diaphragm Muscle Remodeling, Inflammation, Oxidative Stress and Vascularization in Smokers: An Autopsy Study. Cell. Physiol. Biochem..

[17] Nucci R.A.B., de Souza R.R., Suemoto C.K., Busse A.L., Maifrino L.B.M., Anaruma C.A., Pasqualucci C.A., Jacob-Filho W. (2020). Diaphragm muscle structure in the elderly: Findings from an autopsy study. Acta Histochem..

[18] Greising S.M., Mantilla C.B., Gorman B.A., Ermilov L.G., Sieck G.C. (2013). Diaphragm muscle sarcopenia in aging mice. Exp. Gerontol..

[19] Ottenheijm C.A., Heunks L.M., Dekhuijzen R.P. (2008). Diaphragm adaptations in patients with COPD. Respir. Res..

[20] Yang C.J., Tsai M.J., Hung J.Y., Tsai Y.M., Lee J.Y., Chou S.H., Liu T.C., Shen M.C., Huang M.S., Chong I.W. (2016). Poorer prognosis in Taiwanese female ever smokers with stage IV lung adenocarcinoma who were readministered a tyrosine kinase inhibitor. Onco Targets Ther..

[21] Moon H.J., Lim J.E., Jee S.H. (2017). Association between serum concentrations of persistent organic pollutants and smoking in Koreans: A cross-sectional study. J. Epidemiol..

[22] Notara V., Panagiotakos D.B., Kouroupi S., Stergiouli I., Kogias Y., Stravopodis P., Papanagnou G., Zombolos S., Mantas Y., Antonoulas A. (2015). Smoking determines the 10-year (2004–2014) prognosis in patients with Acute Coronary Syndrome: The GREECS observational study. Tob. Induc. Dis..

[23] Scott A., Wang X., Reid W.D. (2006). Increased injury and intramuscular collagen of the diaphragm in COPD: Autopsy observations. Eur. Respir. J..

[24] Nucci R.A.B., Jacob-Filho W., Busse A.L., Maifrino L.B.M., de Souza R.R. (2018). Anatomopathological Assessment of the Diaphragm in Formalin-Fixed, Paraffin-Embedded Sections. J. Morphol. Sci..

[25] MacCoun R., Perlmutter S. (2015). Blind analysis: Hide results to seek the truth. Nature.

[26] Ten V.S., Pinsky D.J. (2002). Endothelial response to hypoxia: Physiologic adaptation and pathologic dysfunction. Curr. Opin. Crit. Care.

[27] Rom O., Kaisari S., Aizenbud D., Reznick A.Z. (2012). Sarcopenia and smoking: A possible cellular model of cigarette smoke effects on muscle protein breakdown. Ann. N. Y. Acad. Sci..

